# Phlebotomines (Diptera: Psychodidae) in a Hydroelectric System Affected Area from Northern Amazonian Brazil: Further Insights into the Effects of Environmental Changes on Vector Ecology

**DOI:** 10.1155/2016/9819723

**Published:** 2016-11-29

**Authors:** Nercy Virginia Rabelo Furtado, Allan Kardec Ribeiro Galardo, Clicia Denis Galardo, Viviane Caetano Firmino, Thiago Vasconcelos dos Santos

**Affiliations:** ^1^Laboratório de Entomologia Médica, Instituto de Pesquisas Científicas e Tecnológicas do Estado do Amapá, Macapá, AP, Brazil; ^2^Universidade Federal do Amapá, Campus Binacional, Oiapoque, AP, Brazil; ^3^Instituto Evandro Chagas, Secretaria de Vigilância em Saúde, Ministério da Saúde, Ananindeua, PA, Brazil

## Abstract

During 2012–2015, an entomological survey was conducted as part of a phlebotomine (Diptera: Psychodidae) monitoring program in an area influenced by the Santo Antônio do Jari hydroelectric system (Amapá State, Brazil). The purpose was to study aspects of Amazon/Guianan American cutaneous leishmaniasis (ACL) vectors subjected to stresses by anthropogenic environmental changes. For sampling, CDC light traps were positioned 0.5, 1, and 20 m above ground at five capture locations along the Jari River Basin. Fluctuations in phlebotomine numbers were analyzed to determine any correlation with rainfall, dam waterlogging, and/or ACL cases, from May 2012 to March 2015. We captured 2,800 individuals, and among 45 species identified,* Bichromomyia flaviscutellata*,* Nyssomyia umbratilis*, and* Psychodopygus squamiventris s.l.* were determined to be the main putative vectors, based on current knowledge of the Amazon/Guianan ACL scenario. Rainfall, but not complete flooding, was relatively correlated with phlebotomine fluctuation, mainly observed for* Ps. squamiventris s.l.*, as were ACL cases with* Ny. umbratilis. *Behavioral changes were observed in the unexpected high frequency of* Bi. flaviscutellata* among CDC captures and the noncanopy dominance of* Ny. umbratilis*, possibly attributable to environmental stress in the sampled ecotopes. Continuous entomological surveillance is necessary to monitor the outcomes of these findings.

## 1. Introduction

Insects of Phlebotominae (Diptera: Psychodidae) species are of great medical interest because of their implications in the transmission of some vector-borne diseases, in particular leishmaniasis [1–3]. American cutaneous leishmaniasis (ACL) is known to be endemic in Amazonian Brazil and is regarded as a serious risk to public health. The high biodiversity of the region induces a complex mosaic of ecological interactions among reservoirs, vectors, and parasites, and consequently a wide spectrum of ACL clinical manifestations, including disfiguring and potentially life-threatening cases [4]. Moreover, this diversity of transmission cycles becomes even more complex to understand in the context of the influence of anthropic effects on natural ecological systems. The consequences of anthropogenic environmental stress on the expansion of the geographical distribution of ACL are, therefore, difficult to predict with any certainty [5].

Over the past 30 years, one of the main causes of human influence on the environment in the Brazilian Amazon Basin is its exploitation for generating energy from hydropower. Negative effects of some of the resultant environmental changes have been observed; an example is the disturbance caused by the proliferation of* Mansonia* (Diptera: Culicidae) in the dammed lake associated with the Tucuruí hydroelectric system [6]. Vector monitoring programs, which are essential to being granted a license to operate, are included in environmental and public health planning, in order to predict and minimize the effects of vector-borne diseases in hydroelectric systems [7]. This monitoring includes spatial/temporal studies of phlebotomines and their potential implications for the transmission dynamics of leishmaniasis agents [8].

The geographically closely related Brazilian states of Pará (PA) and Amapá (AP) share a border through the Jari River and cover a region historically associated with the exploitation and processing of cellulose. Early information about leishmaniasis in the region first emerged in the 1970–1980s. In this period, studies conducted in “Monte Dourado” (Almeirim, PA) provided substantial knowledge about ACL ecoepidemiology, with particular reference to the “pian-bois,” a form of cutaneous leishmaniasis caused by* Leishmania *(*Viannia*)* guyanensis* (formerly referred to as* Leishmania braziliensis guyanensis*), and its respective identity-obscured vector,* Nyssomyia umbratilis* [9–16].

Since those original studies were undertaken, the region also now hosts the Santo Antônio do Jari hydroelectric system. The present study, therefore, aimed to characterize phlebotomine fauna in the area in order to assess the potential impact of the Santo Antônio do Jari hydroelectric system and in particular the spatiotemporal implications of environmental changes on the ecology of ACL vectors in that region.

## 2. Materials and Methods

### 2.1. Study Area

The Santo Antonio do Jari hydroelectric system (0°39′02.4′′S 52°30′56.9′′W) is situated between the municipalities of Almeirim (PA) and Laranjal do Jari (AP), a region within the Guianan Ecoregion Complex. The hydroelectric system comprises 373.4 MW of installed potency and a reservoir area of 31.7 km^2^. Its construction began in 2011, and the system was totally operative from 2014.

The Amazon Region has a “hot-humid” equatorial climate; however, only minor differences are observed in the distinct ecoregions. In this study, four Guianan Ecoregion Complex seasons were characterized as follows: a short rainy (wet) season from mid-November (“novembre humide”) to late January; a short dry season between early February and mid-March; a long rainy season from late March to late July; and a long dry season from late July to mid-November [17].

### 2.2. Sampled Locations

**Figure 1:**
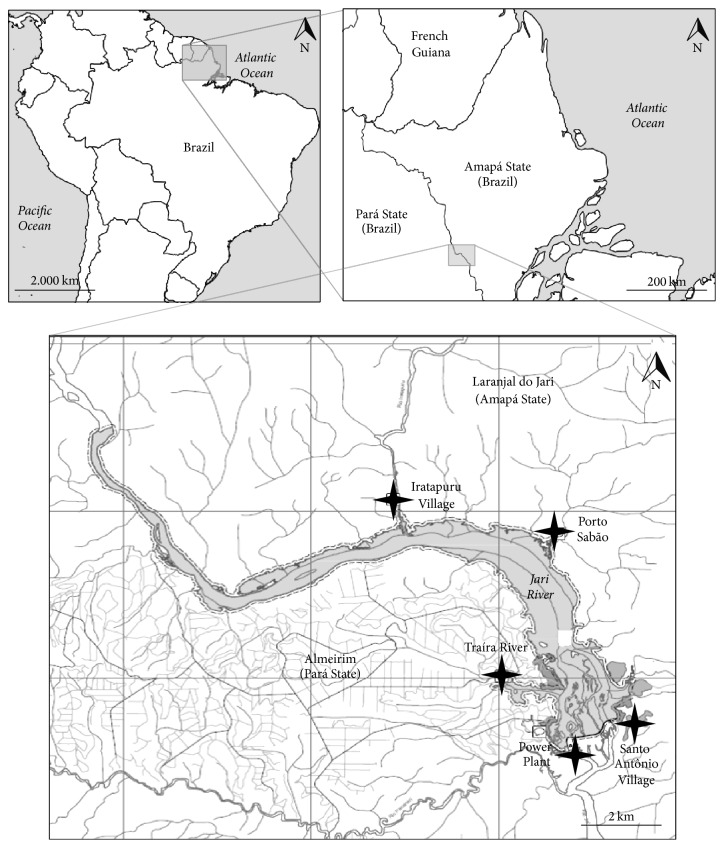
Study area. Maps showing the five surveyed locations for phlebotomine captures (2012–2015) in Santo Antônio do Jari hydroelectric system impacted area, between the states of Pará and Amapá, Brazil. *✦*: locations.

Five locations, each with the following characteristics, were sampled up- and downstream of the caisson (Figure 1):

The eastern bank of the Jari River (AP), covered by alluvial dense forest:

- Porto Sabão (S 00°37′02.2′′ W 052°30′42.1′′), a forested area with only a few dwelling. A small harbor operates from the opposite side of the river.
- Iratapuru Village (S 00°33′50.9′′ W 052°34′48.5′′), a cluster of dwellings, forming a community of approximately 200 inhabitants, which primarily acts as an extractive reserve.
- Santo Antônio Village (S 00°38′57.4′′ W 052°30′27.9′′), which is the only site downstream of the caisson, comprising 27 agriculturist families inhabiting riverine wooden buildings.
- The western bank of the Jari River (PA), covered by submontane (lowland) dense forest.
- Traíra (S 00°37′50.7′′ W 052°31′48.8′′), a location with no dwellings on the Traíra river.
- the power Plant (S 00°39′07.7′′ W 052°31′06.4′′), the most affected area, close to the construction site and with very little vegetation.

### 2.3. Sampling

Phlebotomine captures were undertaken during 2012 to 2015 in fourteen quadrimonthly field expeditions, which aimed to sample periods in the short dry (February/March), rainy to dry (June/July), and dry to rainy (October/November) Amazon/Guianan seasons. Eight expeditions were undertaken before and six after dammed lake levels were replenished (in May 2014). In each of the above described five areas, a randomly selected horizontal transect was established in the direction of the primarily forested area, comprising a phlebotomine monitoring station (MS), sited approximately 200 m distance from the forest edge, where human settlements were present. Each MS incorporated three CDC miniature light traps (Sudia and Chamberlain model), installed between 18:00 and 06:00 for two consecutive nights. Each was placed according to the following different vertical* strata*: 0.5 m from ground level, 1 m from ground level, and at 20 m, in the tree canopy; they were placed 10 m apart from each other. Trees used for canopy placement of CDC traps were selected based on a minimum height of 20 m, regardless of species. The ground level surface was composed of humid, decomposed organic material, originating from the trees. Phlebotomines were triaged in a stereomicroscope, cleaned with neutral detergent [1% (Extran®)], washed with NaCl 0.9%, and then stored in 70% ethanol. In the laboratory, specimens were processed according to the methodology of Ryan [18], mounted in Berlese fluid (GBI Laboratories, Manchester, England), and identified according to Galati [19].

### 2.4. Data Analysis

Sampling effort was calculated based on number of expeditions, versus the number of installed CDC traps, versus hours of exposition. Phlebotomine frequency was analyzed for correlation with climatic data (monthly average rainfall, temperature, and air relative humidity) sourced from the Jari Meteorological Station (“Jari Energia” database) and with epidemiology (ACL monthly prevalence) obtained after interrogating the database of the “Coordenação de Vigilância em Saúde” (Coordination of Surveillance in Health) of the “Secretaria de Estado da Saúde do Amapá-SESA” (Amapá State Health Secretary). Data were tested by a single linear regression with Pearson's coefficient (*r*), using BioEstat 5.0, where the* r-*value represents a strong correlation if above 0.7, a moderate correlation if between 0.3 and 0.7, a weak correlation if between 0.1 and 0.3, and an insignificant correlation if below 0.1. The index of species abundance (ISA) and the standard index of species abundance (SISA) were calculated using Microsoft Excel for the five ecotopes surveyed, as described by Roberts and Hsi [20]. Species diversity (Shannon's diversity index,* H*′) and the coefficient of variation (*S*′) between monthly records (climatic and epidemiological) were calculated using Past software [21]. All comparisons were performed using a* t-*test to determine significance level (*p* value ≤ 0.05).

## 3. Results

A total of 2,800 individuals were captured (1,894 females and 906 males), belonging to 45 species, in approximately 2,520 h of sampling effort. Females of* Trichophoromyia *(exceptionally* Th. ubiquitalis*) and* Pressatia *(not identifiable at species level), and some unidentified* Evandromyia*,* Nyssomyia*, and* Psychodopygus* species were allocated at generic level (Table 1).

New AP records included* Th. castanheirai*,* Th. eurypyga*,* Evandromyia begonae*,* Ev. bacula*,* Pressatia equatorialis*, and* Psathyromyia punctigeniculata*.

The five most frequent species captured were* Sciopemyia sordellii* (*n* = 316; SISA = 0.87),* Ps. davisi *(*n* = 314; SISA = 0.74)*, Bichromomyia flaviscutellata* (*n* = 265; SISA = 0.95)*, Ev. infraspinosa *(*n* = 150; SISA = 0.6), and* Ny. umbratilis *(*n* = 146; SISA = 0.63). Less frequently captured but nonetheless epidemiologically relevant species included* Ps. squamiventris s.l.* (*n* = 89; SISA = 0.52),* Th. ubiquitalis* (*n* = 86; SISA=0.5),* Ps. hirsutus hirsutus* (*n* = 55; SISA = 0.4),* Ny. anduzei* (*n* = 45; SISA = 0.23),* Lutzomyia gomezi* (*n* = 38; SISA = 0.12),* Ny. antunesi* (*n* = 19; SISA = 0.37),* Ps. paraensis* (*n* = 15; SISA = 0.2), and* Ny. whitmani* (*n* = 8; SISA = 0.09).

**Figure 2:**
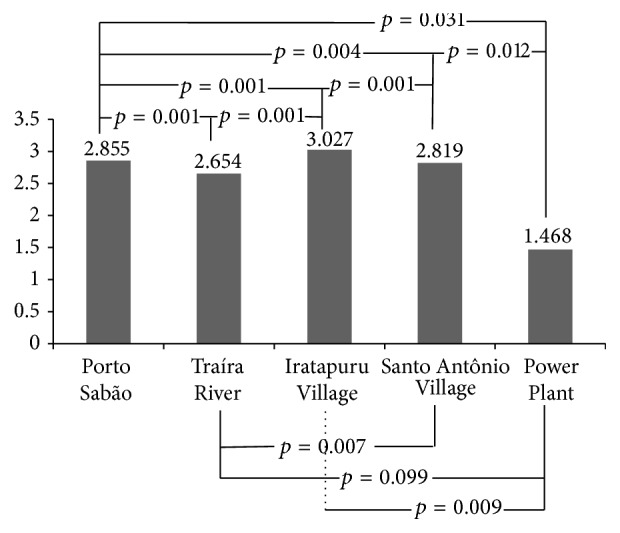
Comparison of Shannon diversity indexes for CDC-captured phlebotomines from the five surveyed locations in Santo Antônio do Jari hydroelectric system impacted area, between the states of Pará and Amapá, Brazil (2012–2015).

Sampling effort for each area comprised approximately 504 h of CDC trapping (14 expeditions, 3 CDC traps, and 12-hour CDC exposition). Porto Sabão was the site of the most captured specimens (1,267), followed by Santo Antônio Village (601). The highest diversity was observed in captures from Iratapuru Village (*H*′ = 3.027). All *H*′ value comparisons between the different environments were significant (*p* ≤ 0.05), except when comparing Traíra River and the Power Plant (*p* ≤ 0.099) (Figure 2).

Regarding vertical distribution, sampling of the same captures described above accounted for approximately 840 h of CDC trapping by* stratum* (14 expeditions, 5 ecotopes, and 12-hour CDC exposition). CDC traps placed 0.5 m above ground level accounted for a greater number of individuals (1,225) than those placed 1 m above ground level (1,021) and 20 m above ground level in the canopy (554) (Table 2). While* Ps. davisi* was most likely to be found at canopy level (75 individuals at 0.5 m, 83 at 1 m, and 156 at 20 m) and* Bi. flaviscutellata* closer to ground level (130 at 0.5 m, 120 at 1 m, and 15 at 20 m), there was little difference in numbers of* Ny. umbratilis* individuals captured at the different levels of* strata* (58 at 0.5 m, 39 at 1 m, and 49 at 20 m).

**Figure 3:**
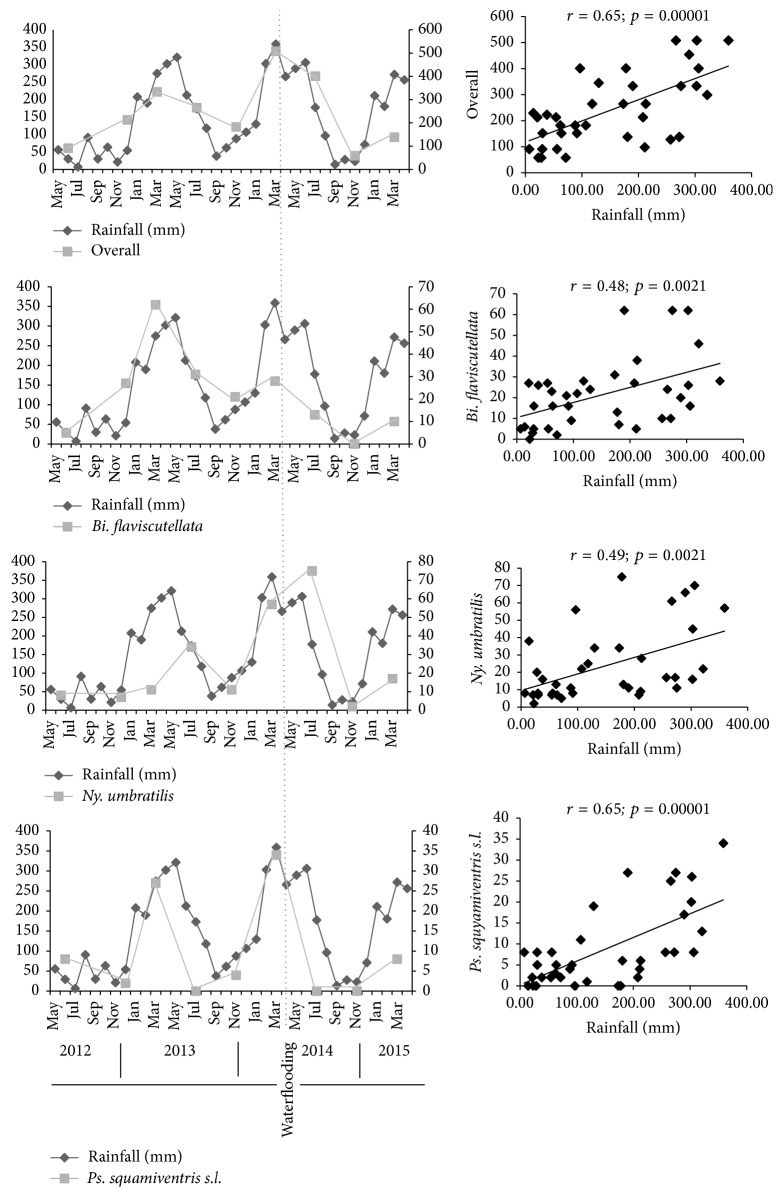
Correlation between rainfall and phlebotomine putative vector species captured in Santo Antônio do Jari hydroelectric system impacted area, between the states of Pará and Amapá, Brazil (2012–2015).

The monthly average rainfall was the only climatic data with a representative coefficient of variance (*S*′ = 70.81) when tested against ACL occurrence and phlebotomine frequency (*S*′ = 65.95), in contrast to temperature (*S*′ = 5.96), and relative air humidity (*S*′ = 9.56). The available data enabled correlations to be determined between the periods of May 2012 to April 2015, a period within which nine of the fourteen capture efforts were undertaken. Rainfall presented a moderate correlation with overall phlebotomine frequency (*r* = 0.65; *p* = 0.00001), as also individually compared with* Ps. squamiventris s.l.* (*r* = 0.65; *p* = 0.00001), and a similarly moderate, but lower correlation with* Bi. flaviscutellata* (*r* = 0.48; *p* = 0.0021) and* Ny. umbratilis* (*r* = 0.48; *p* = 0.002). A decrease in the overall phlebotomine population was observed after March until November 2014, coinciding with the transition between the rainy and dry periods and with flooding (Figure 3).

**Figure 4:**
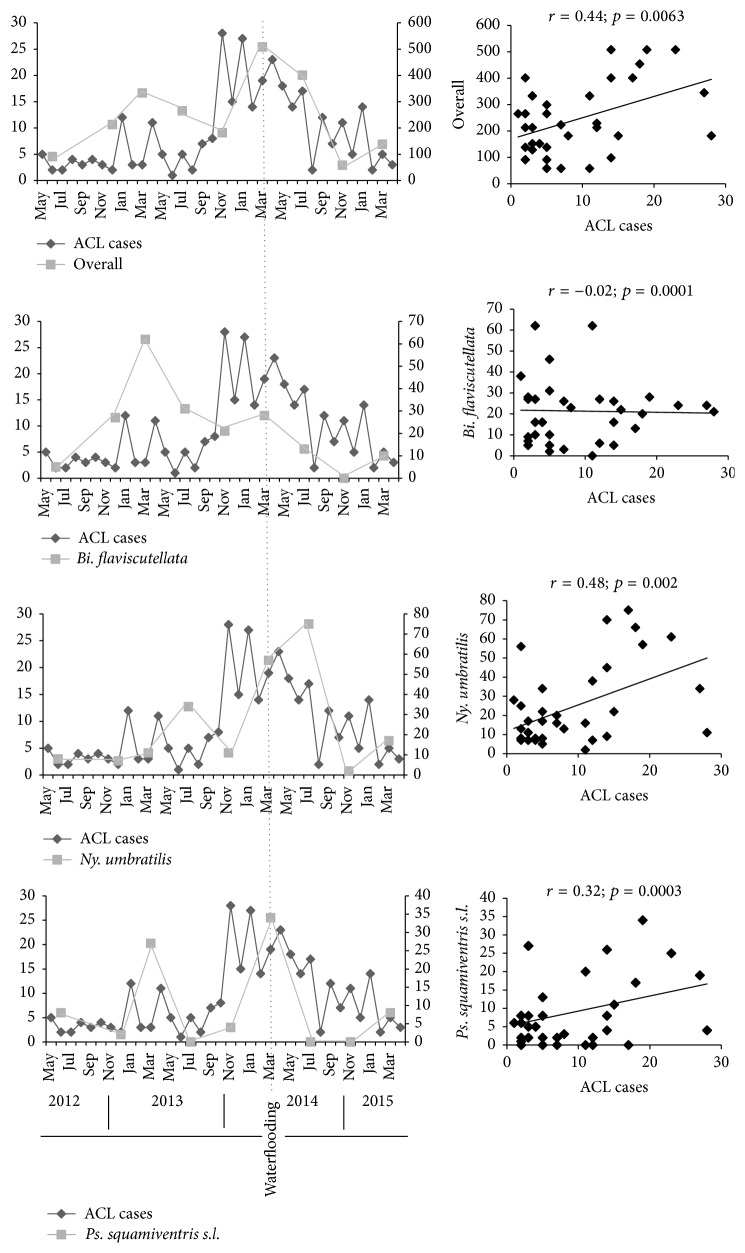
Correlation between ACL cases and phlebotomine putative vector species captured in Santo Antônio do Jari hydroelectric system impacted area, between the states of Pará and Amapá, Brazil (2012–2015).

The number of ACL cases presented a moderate correlation with overall phlebotomine frequency (*r* = 0.44; *p* = 0.0063) and also with* Ps. squamiventris *(*r* = 0.32; *p* = 0.0003) and an insignificant negative correlation with* Bi. flaviscutellata* (*r* = −0.02; *p* = 0.0001). A similarly moderate, but higher correlation was however found with* Ny. umbratilis* (*r* = 0.48; *p* = 0.002) (Figure 4).

## 4. Discussion

It is important to consider current investigations of phlebotomine fauna in the Jari River Basin in the context of the historical findings from the 1970s and 1980s. These earlier studies represent comprehensive research conducted in Monte Dourado (northern PA), a region close to the area investigated in the present study and on which the present epidemiological background to ACL transmission in the associated region is based. In brief, the previous research includes a description of* Ny. umbratilis* (formerly “obscured” as* Ny. anduzei*) [13] and its incrimination as the main vector of* L. *(*V.*)* guyanensis* [9–11], confirmed by strong ecological associations [15]. Just as important, a parasite, more recently described as* L. *(*V.*)* naiffi*, was isolated from the armadillo,* Dasypus novemcinctus, *for which transmission was locally driven and attributed to* Ps. squamiventris s.l.*, and other* Psychodopygus* species [22]; this comprised a primarily enzootic ACL, occasionally infecting humans [23]. Finally, the isolation of* L. *(*L.*)* amazonensis* from rodents in an area affected by occurrence of the main vector* Bi. flaviscutellata* [11] comprised the third known Jari ACL transmission cycle. These facts described above suggest that* Ny. umbratilis*,* Ps. squamiventris s.l.*, and* Bi. flaviscutellata *are implicated in the transmission scenario of the area covered by the present study and thus merit particular attention.

The present study incorporated a representative composition of the known AP fauna. Considering the substantial sampling effort (2,520 h), the determined diversity showed agreement with the findings of other inventories from the Brazilian Amazon basin [1, 24]. Moreover, all new records for AP have already been recorded for PA [25].

Apart from* Ny. umbratilis*,* Ps. squamiventris s.l.*, and* Bi. flaviscutellata, *other species captured were noted as being epidemiologically relevant to ACL in the Brazilian Amazon Region:* Ps. paraensis*,* Ps. davisi*, and* Ps. hirsutus hirsutus*, which are putative vectors of* L. *(*V*.)* naiffi* [26, 27];* Th. ubiquitalis,* a proven vector of* L. *(*V*.)* lainsoni* [28];* Ny. anduzei, *a secondary vector of* L. *(*V.*)* guyanensis *[4];* Ny. whitmani *and* Lu. gomezi,* proven and putative vectors of* L. *(*V*.)* shawi*, respectively [4, 26]; and lastly,* Ny. antunesi, *a suspected vector of* L. *(*V.*)* lindenbergi* [4].

Among the above species,* Ps. davisi* is notable as the third highest ranking according to SISA (0.74) and as having the highest presence in all vertical* strata*, in particular, at the canopy level. This species is generally anthropophilic, is widely distributed in the Amazon Basin [1, 24], and is related to* Leishmania* (*Viannia*) infections in the States of Rondônia (RO) [29] and PA [27, 30]. Interestingly, this species remains frequent in recently surveyed areas affected by hydroelectric schemes in RO [8] and PA (TVS, unpublished). These observations add weight to the need for further investigation into the putative epidemiological role of* Ps. davisi* in Jari.

The species most frequently captured,* Sc. sordellii*, would appear not to be of epidemiological concern. Firstly, the apparent abundance of a given phlebotomine species is not sufficient of itself to incriminate it as a vector [31], particularly when it does not demonstrate high levels of anthropophilic behavior. Secondly, flagellate infections isolated from this species (formerly referred to as* Lutzomyia nordestina*) are historically recognized as distinct from* Leishmania* [22] or remain undetermined, [32] and are more likely to be other trypanosomatids [22]. Furthermore, despite recent reports of molecular* Leishmania* ascribed to this fly [33, 34], currently, polymerase chain reaction-based techniques do not allow “true infection” to be distinguished from a “simple and occasionally ingested” DNA fragment [3]. Thus, there remains insufficient evidence for a potential vector role of* S. sordellii *in* Leishmania *transmission.

High frequencies of light-trapped* Bi. flaviscutellata* have been documented [8, 35], despite rodent-baited trapping being better recognized as a method for attracting the species. Additionally, the first SISA Rank (0.95) was indicative of this high presence in all five surveyed ecotopes; this has been observed in the Belém metropolitan area (PA), where* Bi. flaviscutellata* was the only omnipresent species in seven urban and ecologically isolated forest fragments surveyed [36]. Furthermore, this species has been reported as being able to adapt to plantations of introduced tree species and in other nonclimax forests in Jari [14], suggesting it is capable of responding positively to environmental change, such as the vegetal suppression and flooding caused by hydroelectric operations. Nonetheless, ecological niche model projections predict no change in spatial distribution for* Bi. flaviscutellata* in the Jari area, which is an environment that remains suitable for the species [37].

In an area in Regina, French Guiana, ecologically disturbed due to agricultural practices, flagellates infecting a* Bi. flaviscutellata* specimen have been determined to be* L. *(*V.*)* guyanensis* [38], in similar circumstances to the observed low presence of* Ny. umbratilis*. Although this possibly changing pattern in ACL transmission should be interpreted with caution, it raises an intriguing possibility about the permissiveness of* Bi. flaviscutellata* to* L. *(*V.*)* guyanensis* in the Amazon/Guianan ACL scenario.

In the present study, a higher number of* Bi. flaviscutellata* individuals was captured compared to* Ny. umbratilis *(265 versus 146, resp.). A lack of available evidence for natural infection in this unnatural vector/parasite system, however, precludes speculation about the possible involvement of* Bi. flaviscutellata*, even in the context of occasional transmission of parasites distinct from* L. *(*L.*)* amazonensis.* Nevertheless, the current high occurrence of* Bi. flaviscutellata *in the Amazon/Guianan ACL scenario may be of future concern; unnatural parasite/vector/reservoir systems, when closely related in an ecological context, can, on a century-level scale, undergo stress adaptation (evolutionary fitness) and form new ecological systems driven by coevolution [39].

There is no doubt that* Ny. umbratilis* is the main vector of ACL in the Jari River Basin [15]. Surprisingly, the well-documented tree-dwelling behavior of this species [15, 40] was not observed in CDC canopy captures in the present study; very little difference was found between the number of individuals captured in the three sampled* strata* (58 at 0.5 m, 39 at 1 m, and 49 at 20 m). Although* Ny. umbratilis* is known to climb into the canopy at night to feed on edentates (mainly sloths) [12, 41], it is possible that an alternative terrestrial source of blood, such as rodents, may be attracting females toward ground level. Sloths are very slow moving and are therefore commonly rescued for relocation during environmental monitoring operations; their resulting rareness means* Ny. umbratilis* has had to find other food sources. Conversely, blood meal analysis conducted during studies in a forest fragment of the Western Amazon has shown rodent blood to be the predominant feeding source for* Ny. umbratilis *[42]. Wild and synanthropic rodents have been shown to survive environmental disturbance in Jari and to proliferate in areas of introduced tree species [14]; this indicated a plausible reason not only for the putative adaptation of* Ny. umbratilis*, but also for the increasing population levels of the rodent blood-feeder* Bi. flaviscutellata,* as already discussed.

Given the clear importance of* Psychodopygus *of the Chagasi series (formerly referred to as the Squamiventris series) in the context of ACL transmission [22], taxonomic studies on identifying the cryptic females have been undertaken previously [43] and more recently, with interesting results (Rodrigo E. Godoy, unpublished). These techniques, however, could not be employed in the field at the time. Early consideration of the assumed geographic distribution of the complex* Ps. squamiventris s.l.* [1, 44] shows that individuals from AP are likely to be predominantly* Ps. s. maripaensis*. Determining males of the species present could clarify these taxonomic uncertainties in the area covered by the present study; however, doubts about identification of some male specimens meant they were grouped as* Ps. squamiventris s.l.*

Ryan et al. [22] did not find* Ps. squamiventris s.l.* to be naturally infected in the Jari area but proposed a link to* L*. (*V*.)* naiffi* along the same northern bank of the Amazon River through studies in Cachoeira Porteira (PA); Fouque et al. [38] came to the same conclusions in French Guiana. Interestingly, this fly species complex was found alongside* L. *(*V.*)* braziliensis *DNA in Suriname [45], an area where the known Amazonian vectors of this parasite,* Ps. complexus *and* Ps. wellcomei*, are unlikely to occur. As previously discussed in the context of* Bi. flaviscutellata*, the changing transmission patterns of unnatural vector/parasite systems must be carefully interpreted and only after strong ecological associations have been determined. Thus, the putative vector importance of* Ps. squamiventris s.l.* in Jari can only be assumed in respect of* L. *(*V.*)* naiffi.*

The five surveyed areas presented, in general, different diversity indexes, showing variations in the phlebotomine composition along the affected area. This finding can be attributed not only to the distance between capture sites, but also to forest composition, fragmentation, geographic barriers, and migration of mammal fauna between sites; this latter can occur naturally or be induced by rescue operations involved in hydroelectric planning, whereby animals are moved to suitable “dry land” environments safe from potential flooding.

A decrease in the phlebotomine population in 2014, in the period after filling the reservoir, could not be attributed exclusively either to flooding or to the transition from the rainy to the dry season. Both eventualities, although opposite in effect, are likely to lead to unsuitable conditions for phlebotomine reproduction. However, they did coincide in May 2014, when rainfall decreased and the lake was formed.

Conversely, rainfall was a valid determinant of overall phlebotomine fluctuation;* Ps. squamiventris s.l.* was the main putative vector directly correlated with this. Other variables, such as forest composition and altitudinal variation, are known to influence seasonal dynamics of species from the complex* Ps. squamiventris s.l.* For example, the dry period from October to November in Serra dos Carajás (southern PA) can be unsuitable for* Ps. wellcomei*, which is adapted to high altitudes, in contrast to* Ps. complexus*, which flourishes in lowland areas during the same months [46].

An insignificant negative to a moderate positive correlation was observed between cases of ACL and phlebotomine fluctuations. In the case of* Ny. umbratilis, *however, the apparently higher correlation when compared with other species is in agreement with its known vector importance in Amazon/Guianan ACL. A strong spatiotemporal association between a given vector and ACL cases is considered essential to maintenance of the* Leishmania* life cycle [2, 14].

In summary, the present study filled a temporal gap on phlebotomine data relating to the Jari area. It provides a meaningful update on species composition and further insights into seasonal dynamics of species under pressures caused by environmental change. The presence of ACL vectors, in particular* Ny. umbratilis*,* Bi. flaviscutellata*, and* Ps. squamiventris s.l.,* is noteworthy, due to their direct association with Amazon/Guianan-ACL transmission. For* Ny. umbratilis*, the lower frequency associated with the unpredicted lack of tree-dwelling behavior may be attributable to a reduction in numbers of canopy-dwelling mammals, due to the environmental disturbance and relocation efforts; this could consequently have led to seeking for alternative terrestrial blood sources at ground level. The possible association of* Ps. davisi* with* Leishmania *transmission cycles in other Amazon ecoregions draws attention to its high rank position in Jari. Rainfall can be partly associated with local seasonal fluctuations in fly numbers, mainly with respect to* Ps. squamiventris s.l.* Cases of ACL were also more highly correlated with* Ny. umbratilis* dynamics. Further attention was also given to results for* Bi. flaviscutellata*, which has been apparently tolerant of anthropic activity: continued phlebotomine monitoring is proposed in order to evaluate the implication for social welfare and health in the future.

## Figures and Tables

**Table 1:** Phebotomine species composition of Santo Antônio do Jari hydroelectric system impacted area (Amapá State, Amazonian Brazil) on the basis of five surveyed locations (2012–2015).

	Species	Porto Sabão		Traíra River		Iratapuru		Santo		Power Plant		ISA	SISA	Rank	Total	%
						Village		Antônio								
								Village								
		♀	♂	♀	♂	♀	♂	♀	♂	♀	♂					
1	*Sciopemyia sordellii*	149	66	13	10	15	4	42	16	—	1	3,6	0,87	2nd	316	11,29
2	***Psychodopygus davisi***	46	37	95	56	15	10	38	17	—	—	6,4	0,74	3rd	314	11,21
—	*Trichophoromyia *spp.	177	—	34	—	40	—	17	—	—	—	—	—	—	268	9,57
3	***Bichromomyia flaviscutellata***	76	32	24	14	22	11	52	31	1	2	2	0,95	1st	265	9,46
4	*Evandromyia infraspinosa*	44	10	25	18	3	2	30	18	—	—	9,2	0,6	6th	150	5,36
5	***Nyssomyia umbratilis***	26	12	31	30	19	6	13	9	—	—	8,6	0,63	5th	146	5,21
6	*Trichophoromyia castanheirai*	—	104	—	6	—	14	—	11	—	—	11,2	0,51	9th	135	4,82
—	*Pressatia *spp.	43	—	1	—	9	—	78	—	—	—	—	—	—	131	4,68
—	*Evandromyia *spp.	52	10	4	—	39	11	12	—	—	—	—	—	—	128	4,57
7	*Viannamyia furcata*	44	4	11	5	11	1	30	5	—	—	10,4	0,55	7th	111	3,96
8	***Psychodopygus squamiventris sensu lato***	36	14	15	5	10	—	9	—	—	—	11	0,52	8th	89	3,18
9	*Trichopygomyia trichopyga*	11	16	9	8	9	—	21	12	—	—	7,2	0,7	4th	86	3,07
10	***Trichophoromyia ubiquitalis***	21	6	5	8	14	8	13	9	2	—	11,4	0,5	10th	86	3,07
11	*Evandromyia saulensis*	40	9	5	—	4	4	12	1	—	—	12,8	0,43	11th	75	2,68
12	***Psychodopygus hirsutus hirsutus***	13	—	20	4	14	4	—	—	—	—	13,6	0,4	12th	55	1,96
13	*Trichophoromyia brachypyga*	—	36	—	12	—	2	—	2	—	—	15,2	0,32	14th	52	1,86
14	*Pressatia choti*	—	17	—	3	—	5	—	25	—	—	14,2	0,37	13th	50	1,79
15	***Nyssomyia anduzei***	6	5	24	10	—	—	—	—	—	—	17	0,23	16th	45	1,61
16	***Lutzomyia gomezi***	27	11	—	—	—	—	—	—	—	—	19,4	0,12	22th	38	1,36
—	*Nyssomyia *spp.	—	—	—	1	1	—	11	25	—	—	—	—	—	38	1,36
17	***Nyssomyia antunesi***	—	2	9	—	5	2	—	—	1	—	14,2	0,37	13th	19	0,68
18	*Sciopemyia fluviatilis*	2	1	3	—	9	1	—	—	—	—	18	0,19	18th	16	0,57
19	*Micropygomyia rorotaensis*	4	—	—	—	8	—	2	1	—	—	17,8	0,2	17th	15	0,54
20	***Psychodopygus paraensis***	—	—	—	—	6	3	5	1	—	—	17,6	0,2	17th	15	0,54
21	*Psychodopygus claustrei*	7	1	—	—	—	—	2	—	3	1	15,4	0,31	15th	14	0,50
—	*Psychodopygus *spp.	4	—	2	—	6	—	2	—	—	—	—	—	—	14	0,50
22	*Trichophoromyia eurypyga*	—	8	—	—	—	3	—	1	—	—	18,2	0,18	19th	12	0,43
23	*Psathyromyia aragaoi*	—	—	5	2	—	—	4	—	—	—	18,8	0,15	20th	11	0,39
24	*Evandromyia brachyphalla*	4	3	2	1	1	—	—	—	—	—	19	0,14	21th	11	0,39
25	*Psychodopygus carrerai carrerai*	1	1	—	7	—	—	—	—	—	—	20,2	0,08	26th	9	0,32
26	*Evandromyia begonae*	2	2	—	—	4	—	—	—	—	—	19,6	0,11	23th	8	0,29
27	***Nyssomyia whitmani***	—	—	—	—	—	—	8	—	—	—	20	0,09	25th	8	0,29
28	*Psathyromyia scaffi*	3	2	—	—	2	—	—	—	—	—	19,8	0,1	24th	7	0,25
29	*Pressatia trispinosa*	—	—	—	7	—	—	—	—	—	—	20,4	0,07	27th	7	0,25
30	*Micropygomyia trinidadensis*	7	—	—	—	—	—	—	—	—	—	20,8	0,05	29th	7	0,25
31	*Viannamyia tuberculata*	6	—	—	—	—	—	—	—	—	—	21	0,04	30th	6	0,21
32	*Pintomyia damascenoi*	—	—	—	—	—	—	5	—	—	—	20	0,09	25th	5	0,18
33	*Evandromyia evandroi*	—	—	—	—	4	1	—	—	—	—	20,4	0,07	27th	5	0,18
34	*Psathyromyia lutziana*	5	—	—	—	—	—	—	—	—	—	21,2	0,03	31th	5	0,18
35	*Brumptomia travassosi*	—	—	—	—	—	—	3	1	—	—	19,6	0,11	23th	4	0,14
36	*Evandromyia bacula*	—	—	—	—	1	—	3	—	—	—	20,2	0,08	26th	4	0,14
37	*Evandromyia monstruosa*	—	—	4	—	—	—	—	—	—	—	20,6	0,06	28th	4	0,14
38	*Psychodopygus amazonensis*	—	—	—	—	2	2	—	—	—	—	21	0,04	30th	4	0,14
39	*Psychodopygus corossoniensis*	—	—	—	—	—	2	—	—	—	—	20,6	0,06	28th	2	0,07
40	*Pressatia equatorialis*	—	2	—	—	—	—	—	—	—	—	21	0,04	30th	2	0,07
41	*Psathyromyia inflata*	—	—	—	—	—	—	2	—	—	—	21	0,04	30th	2	0,07
42	*Micropygomyia micropyga*	—	—	—	—	—	—	1	1	—	—	21,4	0,02	32th	2	0,07
43	*Psychodopygus geniculatus*	—	—	—	2	—	—	—	—	—	—	21,8	0	33th	2	0,07
44	*Lutzomyia carvalhoi*	—	—	1	—	—	—	—	—	—	—	20,8	0,05	29th	1	0,04
45	*Psathyromyia punctigeniculata*	—	—	—	—	1	—	—	—	—	—	21,6	0,01	32th	1	0,04
	*Total*	*856*	*411*	*342*	*209*	*274*	*96*	*415*	*186*	*7*	*4*				*2800*	*100,0*
		*1267*		*551*		*370*		*601*		*11*						

Each location comprised a monitoring station (MS) sampled with 0.5, 1, and 20 m CDC traps, providing approximately 504 h/MS.

♀: female; ♂: male; ISA: index of species abundance; SISA: standard index of species abundance.

“the bold italic font” Proven and/or putative ACL vectors in the Amazon Region, based in Young and Duncan [1], Rangel and Lainson [4], Ready [2], and Lainson and Shaw [16].

**Table 2:** Vertical stratification of Phebotomine species composition of Santo Antônio do Jari hydroelectric system impacted area (Amapá State, Amazonian Brazil) on the basis of five surveyed locations (2012–2015).

	Species	0.5 m ground		1 m ground		20 m canopy		Total
		♀	♂	♀	♂	♀	♂	
1	*Sciopemyia sordellii*	117	71	91	23	11	3	*316*
2	*Psychodopygus davisi*	48	27	44	39	102	54	*314*
—	*Trichophoromyia *spp.	117	—	127	—	24	—	*268*
3	*Bichromomyia flaviscutellata*	85	45	79	41	11	4	*265*
4	*Evandromyia infraspinosa*	38	18	48	27	16	3	*150*
5	*Nyssomyia umbratilis*	35	23	26	13	28	21	*146*
6	*Trichophoromyia castanheirai*	—	50	—	59	—	26	*135*
—	*Pressatia *spp.	66	—	39	—	26	—	*131*
—	*Evandromyia *spp.	55	12	41	9	11	—	*128*
7	*Vianamyia furcata*	42	8	46	6	8	1	*111*
8	*Psychodopygus squamiventris s.l.*	27	11	20	1	23	7	*89*
9	*Trichopygomyia trichopyga*	18	20	10	5	22	11	*86*
10	*Trichophoromyia ubiquitalis*	28	15	16	13	9	5	*86*
—	Other species (11–45)	137	112	129	69	71	57	*575*
	*Total*	813	412	716	305	362	192	*2800*

	*Total* (♀ + ♂)	*1225*		*1021*		*554*		—

Each *stratum* was sampled with approximately 840 h of CDC trap.

♀: female; ♂: male.
